# Receptor-interacting serine/threonine kinase 1- and 3-dependent inflammation induced in lungs of chicken infected with *Pasteurella multocida*

**DOI:** 10.1038/s41598-020-62042-7

**Published:** 2020-04-14

**Authors:** Weitian Li, Qiyu Tang, Na Dai, Weikuan Feng, Changqing Xie, Guofu Cheng, Xiaoli Liu, Wanpo Zhang, Xueying Hu, Changqin Gu

**Affiliations:** Department of Basic Veterinary Medicine, College of Veterinary Medicine, Huazhong Agricultural University, Wuhan, Hubei 430070 China

**Keywords:** Bacterial infection, Acute inflammation

## Abstract

Fowl cholera is a serious, highly contagious disease caused by the bacterium *Pasteurella multocida* (*P. multocida*) in a range of avian species and is characterized by an acute form of septicaemia. The pathogenic mechanism of chicken lung injury caused by the bacterium is unclear. Therefore, *P. multocida* Q (a reference standard strain isolated from chicken) and 1G1 (a clinic isolated strain from duck) were selected to infect chickens, establishing fowl cholera-induced laying hen models. Several important proteins involved in the process of lung injury were identified and quantified using immunohistochemistry and WB. The results showed that chicken lungs infected with bacteria for 24 h showed congestion and edema. The inflammatory factors HMGB1 and IL-6, intercellular matrix MMP, the cell apoptosis-associated caspase-3 and necrotic apoptosis signal molecules RIPK1 and RIPK3 were widely expressed in the lungs of group Q and were significantly different compared with those of 1G1 group and uninfected group (P < 0.05). The results indicated that RIPK1 and RIPK3 are involved in the injury process of chicken lungs after infection with *P. multocida*, and the mechanisms of lung injury induced by different strains are different.

## Introduction

Fowl cholera, a major bacterial disease in poultry, has resulted in substantial economic losses in the chicken industry. In the southern provinces of China, the incidence of fowl cholera is 10–70%, and the mortality rate can reach 30–80%^1^. *Pasteurella multocida* (*P. multocida*), which causes fowl cholera, induces symptoms of acute septicaemia and a range of gross lesions in the lung, eventually leading to death^2–6^. Many studies have investigated the pathogenesis of fowl cholera^7,8^. However, the host immune response to *P. multocida* is still unclear, particularly the inflammation-related molecular mechanisms. Moreover, mice, rather than chickens, are often used as animal models to study the molecular pathogenesis of *P. multocida*^9^. Therefore, further studies are needed to elucidate this issue.

Lung inflammation is the major pathological change observed in fowl cholera. Heterophils are important in this process, initially causing tissue damage and then removing the bacteria to control infection^10^. Inflammation typically results in cell death^11,12^, including apoptosis and necroptosis, which are the two major forms of cell death during infection^13^. Apoptosis is a caspase-dependent cell death signalling pathway, and caspase-3 is a key enzyme involved in this process. Many extracellular apoptotic signals activate caspase-3 and inactivate cytoplasmic, nuclear, and cytoskeletal proteases, causing apoptosis. Necroptosis is a form of regulated necrosis that is executed by receptor-interacting serine/threonine kinase (RIPK) 1 and/or RIPK3 when caspases are inhibited. The receptor-interacting protein (RIP) family plays an important role in regulating apoptosis, programmed necrosis and survival pathways^14^. RIPK1/caspase-3 induces apoptosis, and RIPK1/RIPK3 induces necroptosis. Typically, RIPK1 and RIPK3 complex assembly (necrosome) is key in initiating necroptosis^15–18^. When cellular FLICE (Fas-associating protein with death domain-like interleukin-1β-converting enzyme)/caspase-8 inhibitory protein catalytic activity is blocked, interaction of the RHIM (RIP isomorphism) domain in RIPK1 with that in RIPK3 appears to recruit more RIPK3, causing intramolecular autophosphorylation of RIPK3 and subsequent recruitment of mixed-lineage kinase domain pseudokinases (MLKL)^19–22^. Additionally, necroptosis mediates host defence against pathogens^23^. Therefore, the localization and expression levels of caspase-3, RIPK1, RIPK3, and MLKL may be important in the pathogenesis of *P. multocida*.

Lipopolysaccharide (LPS) is a toxin produced by *P. multocida* that can induce RIPK1 and RIPK3 kinase-dependent inflammation^24^, and high mobility group box 1 (HMGB1) is an important pro-inflammatory factor that promotes both early and late inflammation and modulates non-physiological cell death^25,26^. Monocytes and dendritic cells secrete interleukin (IL)-6 under HMGB1 stimulation^27,28^. Moreover, *P. multocida* virulence factors induce IL-6 in fibroblasts without enhancing IL-1α or TNF-α expression^29^. Matrix metalloproteinase (MMP) 9 is an important factor in neutrophils (heterophils in chickens) and is released during the elimination of infection. MMP9 causes tissue damage and is induced, along with tissue inhibitor of metalloproteinases (TIMPs), by *P. multocida* infection in the lung tissues of mice^30^.

We established a chicken model of *P. multocida* infection to examine the mechanism by which the host resists infection, focusing on the type of cell death that causes lung tissue lesions in chickens and the inflammatory process in the lungs of chickens with cholera.

## Results

### Bacterial identification and serotyping

**Figure 1:**
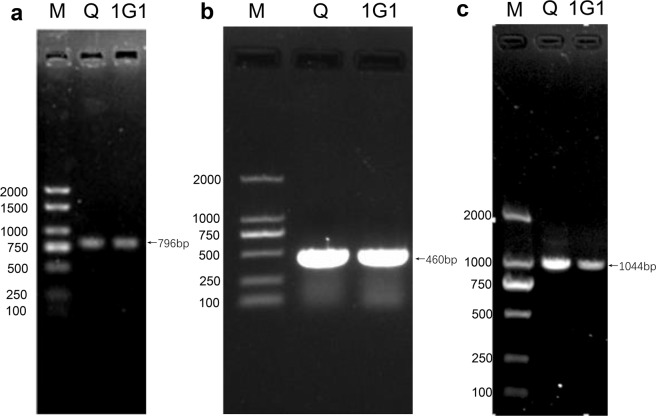
Identification and serotyping of *P*. *multocida* by PCR. (**a**,**b**) PCR amplification of *P*. *multocida* 16S rRNA and KMT1 genes. (**c**) PCR serotyping results. M, DL2000 DNA Marker; Q, Q group; 1G1, 1G1 group.

The results of PCR amplication of 16S ribosomal RNA (rRNA) genes showed that there is a specific band around 796 bp (Fig. 1a). Besides, KMT1 genes amplication PCR assays showed that the target DNA fragments of Q and 1G1 strains were about 460 bp (Fig. 1b), further comfirming both strains were *P. multocida*. The bacterial serotyping PCR assays indicated that the amplified fragments of both strains were around 1000 bp. In light of the target DNA sequences of strains of serotype A known to be 1044 bp (Fig. 1c), Q and 1G1 *P. multocida* strains were classified as serotype A.

### Clinical signs and gross lesions

**Figure 2:**
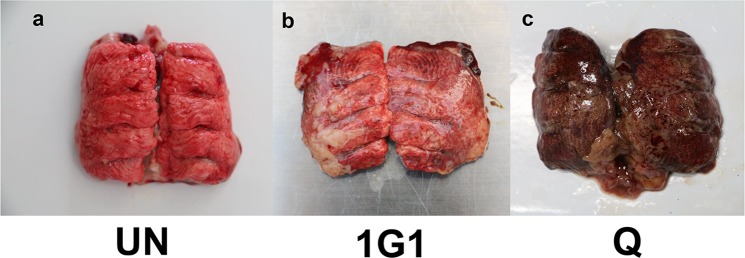
The gross findings from lungs of (**a**) uninfected group, (**b**) 1G1 strain infection group and (**c**) Q strain infection group. (**a**) The lungs of uninfected group showed a healthy state; (**b**) The 1G1 group was presented as the partial congestion of the lung and mild edema; (**c**) The Q strain infected group showed severe hyperemia. The overall color of the lungs is dark red and indicates congestion and edema. UN, uninfected group; Q, Q group; 1G1, 1G1 group.

All chickens inoculated with *P. multocida* showed respiratoryclinical signs 24 h after infection, while chickens in uninfected group appeared normal. After autopsy, the gross lessions of lungs from infected groups (Fig. 2a,b) and uninfected group (Fig. 2c) were carefully observed and recorded. As could be seen from these figures, lungs from uninfected group was in a healthy state (Fig. 2a), while lungs from 1G1 group mainly exhibited slight pathological changes compared with uninfected group, which was represented as partial pulmonary congestion and mild edema (Fig. 2b). Pathological lesions of Q group were presented with more severe congestion and edema compared with 1G1 group. Congestion and edema could be easily identified by dark red lung tissue from gross lesion (Fig. 2c).

### Chicken lung histopathology changes

**Figure 3:**
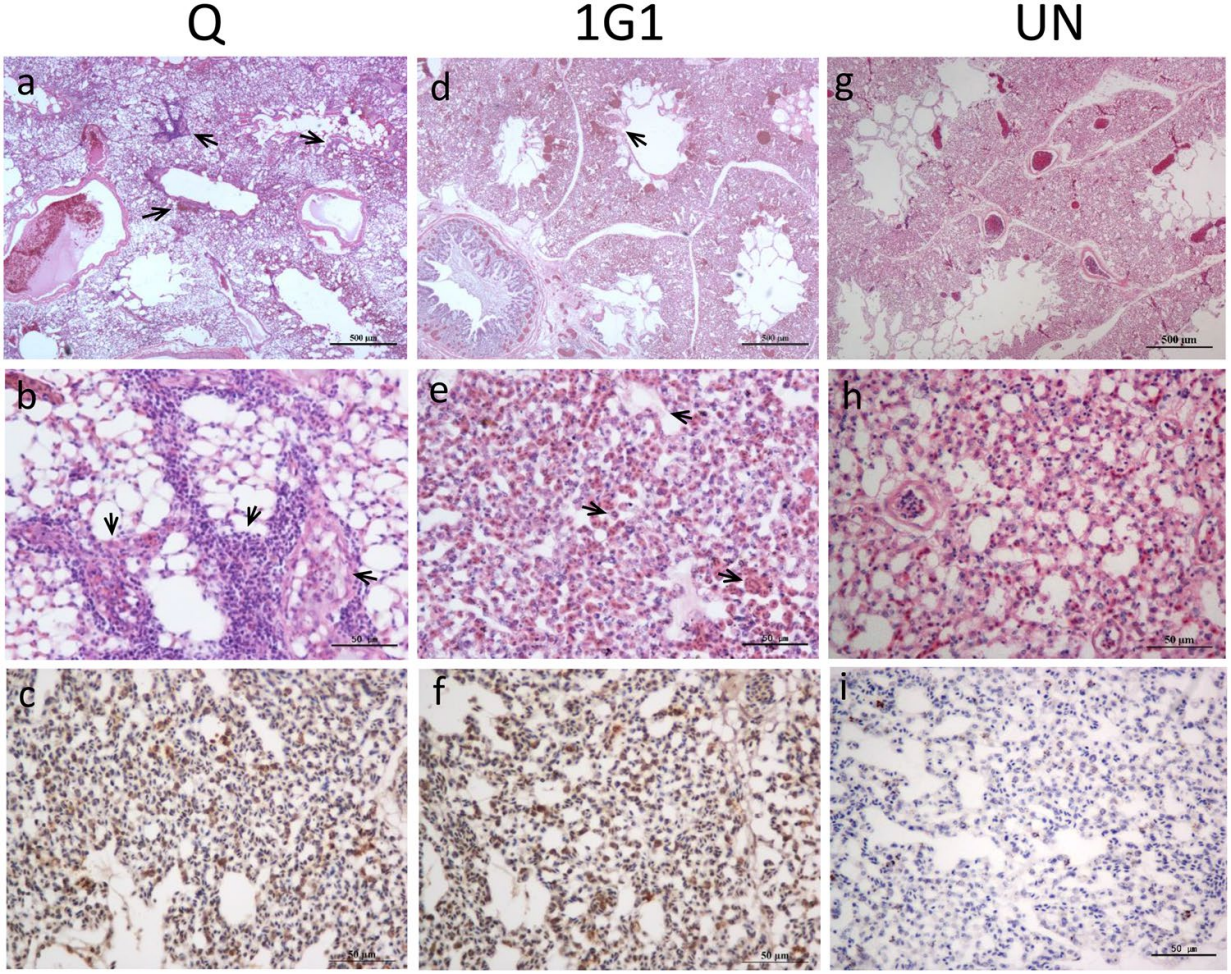
Lung sections from chickens infected with *P. multocida* 24 h post-infection. (**a**,**b**) The lung sections of Q group. (**d**,**e**) The lung sections of 1G1 group. (**g**,**h**) The lung sections of uninfected group. (**c**,**f**,**i**) Immunohistochemical staining of *P. multocida* was performed on sections of lungs from three groups. A large number of *Pasteurella multocida*-specific positive signals could be detected in Q and 1G1 groups. The uninfected group had no positive signals. UN, uninfected group; Q, Q group; 1G1, 1G1 group.

After two bacterial strains infected laying hens, typical pathological changes of lung tissues were confirmed as hyperemia and edema. Specifically, in group Q, pulmonary interstitial capillaries were congested, accompanied by red-stained edema fluid filled in alveolar space. Pulmonary vascular walls became loose, increasing gap with the surrounding tissues. Heterophilic granulocytes infiltrated in alveolar septum and accumulated around small pulmonary vessels (Fig. 3a,b). By contrast, 1G1 group showed a slightly milder pathological changes. Compared with uninfected group, an amount of inflammatory cells appeared in alveolar walls in 1G1 group (Fig. 3d,e). No clear pathological changes were observed in uninfected group (Fig. 3g,h).

Immunohistochemical (IHC) staining of *P. multocida* was performed on sections of lungs from three groups. A large amount of *P. multocida*-specific positive signals could be detected in Q and 1G1 groups, which implies *P. multocida* colonized the lung tissue via the circulatory system (Fig. 3c,f), while the uninfected group had no positive signals (Fig. 3i).

### Expression of related inflammatory factors in lung tissue of chicken after *P. multocida* infection

**Figure 4:**
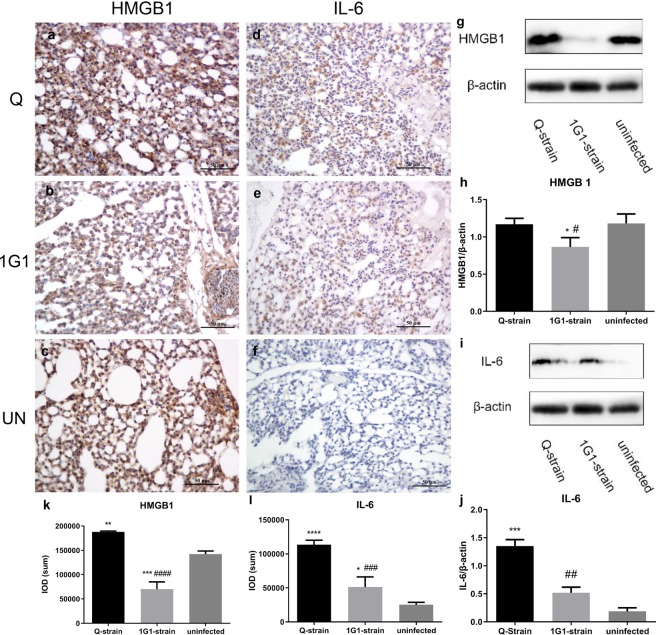
Expression of HMGB1 and IL-6 significantly increased after infection with *P. multocida*. (**a**–**f**) Immunohistochemical results of HMGB1 and IL-6 in three groups. (**k**,**l**) IOD value of Immunohistochemical positive areas of HMGB1 and IL-6. (**g**–**j**) Expression of HMGB1 and IL-6 proteins in chicken lungs from all groups were detected by Western blot. (**g**,**i**) Representative Western blot results of HMGB1 and IL-6. (**h**,**j**) Histogram of expression levels of HMGB1 and IL-6 proteins in the loaded control with β-actin in three groups. UN, uninfected group; Q, Q group; 1G1, 1G1 group. (*P < 0.05; **P < 0.01; ***P < 0.001; ****P < 0.0001, compared with uninfected group; ^#^P < 0.05; ^##^P < 0.01; ^###^P < 0.001; ^####^P < 0.0001, compared with group 1G1; n = 3 per group).

HMGB1 expressed strong positive signals in alveolar surface and nucleus of fibroblasts in uninfected group (Fig. 4c). There were also expressions in cytoplasm of macrophages in 1G1 group (Fig. 4b). In Q group, HMGB1 expression was observed in cytoplasm of fibroblasts, macrophages, heterophilic granulocytes and alveolar cells (Fig. 4a). Then we analyzed IOD (SUM) of HMGB1 positive signals by IHC staining. A comparison of the three groups showed that Q group was significantly different from uninfected group and 1G1 group (Q vs uninfected: P = 0.0026; Q vs 1G1: P = 0.0000). And there was a significant difference between uninfected group and 1G1 group (P = 0.0002; Fig. 4k). The positive area of 1G1 group was less than that of uninfected group. According to a quantitative western blot analysis of HMGB1 (24 kDa), the expression levels of HMGB1 in the three groups were consistent with the difference in the positively stained area by IHC. A significantly lower level was observed in 1G1 group compared with uninfected group and Q group (1G1 vs uninfected: P = 0.0311; 1G1 vs Q, P = 0.0360; Fig. 4h). No significant difference was observed between Q group and uninfected group (P = 0.9920; Fig. 4h).

IL-6 positive signals were not found in lungs of uninfected group (Fig. 4f), but were highly expressed in cytoplasm of heterophilic granulocytes in infected groups (Fig. 4d,e). The positive area IOD (SUM) of 1G1 group was significantly lower than that of Q group (Q vs 1G1: P = 0.0005; Fig. 4l). The difference between infected groups and uninfected group was also significant (Q vs uninfected: P = 0.0000; 1G1 vs uninfected: P = 0.0377; Fig. 4l). The western blot results of IL-6 (23 kDa) confirmed the significant difference between infected groups and uninfected group. Compared with uninfected group, the protein expression level of IL-6 in Q group significantly elevated (P = 0.0003; Fig. 4j), but there was no significant difference between 1G1 group and uninfected group (P = 0.1117; Fig. 4j). And a significant difference between 1G1 group and Q group could be easily observed (P = 0.0021; Fig. 4j).

### Expression of apoptosis-related proteins RIPK1, RIPK3, caspase3 and MLKL in *P.multocida* infected chicken lung

**Figure 5:**
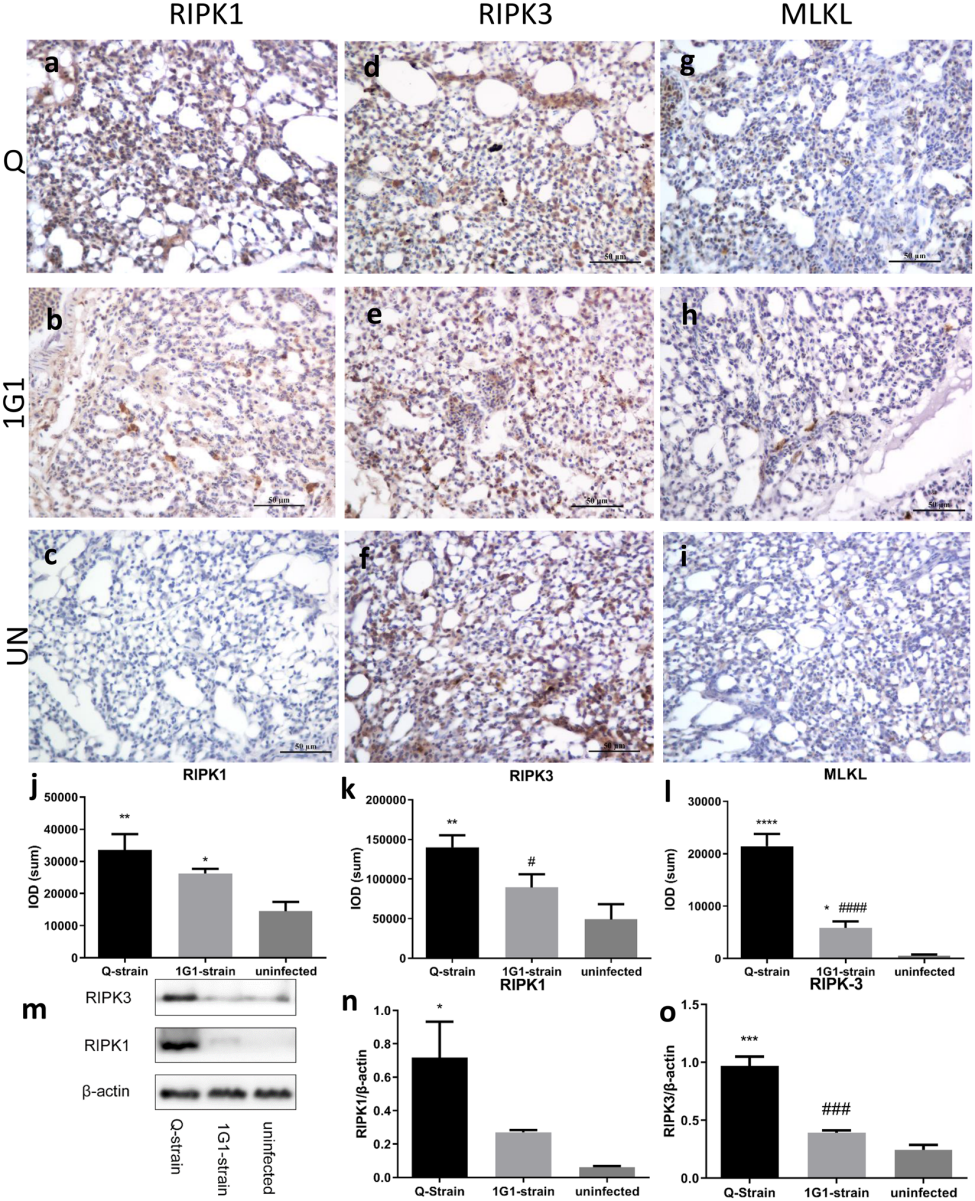
Expression of RIPK1, RIPK3, and MLKL significantly and evidently increased in infected chickens. (**a**) Representative images of immunohistochemistry of RIPK1, RIPK3 and MLKL in lung tissues. (**b**) Comparison of positive areas of RIPK1, RIPK3 and MLKL in infected and uninfected chicken lung tissues. The positive regions of RIPK1 and RIPK3 showed similar increases after infection with *Pasteurella multocida*. (**c**) Western blot RIPK-1 and RIPK3 expression. (**m**) Representative western blot results of RIPK1 and RIPK3 in chicken lung in the three groups. (**n**,**o**) show the ratio of expression of RIPK1 and RIPK3 with β-actin as a control in three groups of chickens in a bar graph. UN, uninfected; Q, Q group; G, 1G1 group. (*P < 0.05; **P < 0.01; ***P < 0.001; ****P < 0.0001, compared with uninfected group; ^#^P < 0.05; ^##^P < 0.01; ^###^P < 0.001; ^####^P < 0.0001, compared with group G; n = 3 per group).

We observed a number of positive RIPK1 signals widely distributed in a variety of cells with no apparent cell specificity in Q group (Fig. 5a). In contrast, RIPK1 positive signals in 1G1 group were only observed in macrophages (Fig. 5b). And there were no positive signals in uninfected group (Fig. 5c). Furthermore, RIPK1 positive-stained region IOD (SUM) of Q group and 1G1 group was significantly different from uninfected group (Q vs uninfected: P = 0.0011; 1G1 vs uninfected:P = 0.0123; Fig. 5j).

The positive signals of RIPK3 were mainly found in cytoplasm of heterophilic granulocytes, macrophages and lymphocytes, while alveolar cells and fibroblasts showed a weak positive signal in uninfected group (Fig. 5f). A similar distribution of RIPK3 positive signals were detected in 1G1 group (Fig. 5e), but in Q group a stronger positive signal was observed in alveolar cells and fibroblasts (Fig. 5d). RIPK3-stained areas IOD (SUM) analysis showed that there was a significant difference between infected groups (P = 0.0261, Fig. 5k), and a significant difference between Q group and uninfected group was found (P = 0.0016, Fig. 5k).

MLKL-positive signals was detected in three groups. In particular, expression of MLKL proteins was only observed in a small fraction of alveolar cells in uninfected group (Fig. 5i), while positive signals were easily observed in infected groups. These positive signals were able to be found in cytoplasm of macrophages in 1G1 group (Fig. 5h) and heterophilic granulocytes, fibroblasts and alveolar cells in Q group (Fig. 5g). MLKL-stained areas IOD(SUM) analysis showed that there was a significant difference between infected groups and uninfected group (Q vs uninfected: P = 0.0000; Q vs 1G1: P = 0.0000; Fig. 5l). And the MLKL positive signial in 1G1 group were more intensive than that of uninfected group (P = 0.0131; Fig. 5l).

To further quantify RIPK1 and RIPK3, we utilized western blot to detect their expression levels in three groups. The expression of RIPK1 (76 kDa) in Q group was significantly different from that in uninfected group (P = 0.0235; Fig. 5n). And there was no significant difference between 1G1 group and the other two groups. (1G1 vs uninfected: P = 0.5058; 1G1 vs Q: P = 0.0995; Fig. 5n). For RIPK3 (56 kDa), a significant difference in RIPK1 expression between Q group and uninfected group or 1G1 group (Q vs uninfected: P = 0.0002; Q vs 1G1: P = 0.0006; Fig. 5o), but there was no significant difference between 1G1 group and uninfected group (P = 0.1966; Fig. 5o).

**Figure 6:**
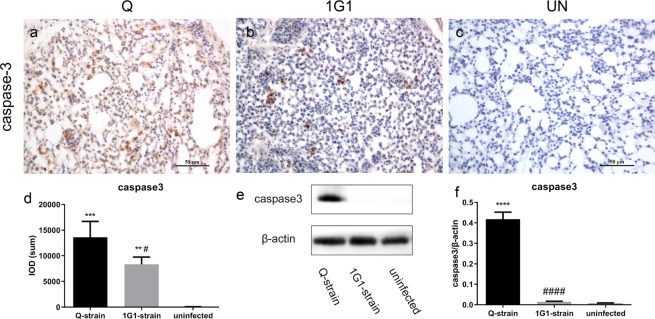
Expression of apoptotic molecule caspase-3 was significantly increased in infected chickens. (**a**) Representative images of caspase-3 immunohistochemistry in lung tissue. (**d**) caspase-3 positive areas in the infected and uninfected groups were compared. (**e**) Representative results of caspase-3 western blot in lungs of three groups of chickens. (**f**) Bar graph of caspase-3 expression in chicken lungs with β-actin as a control in three groups of chickens. UN, uninfected; Q, Q group; G, 1G1 group. (*P < 0.05; **P < 0.01; ***P < 0.001; ****P < 0.0001, compared with uninfected group; ^#^P < 0.05; ^##^P < 0.01; ^###^P < 0.001; ^####^P < 0.0001, compared with group G; n = 3 per group).

In Q group, a positive signal of caspase-3 was observed in cytoplasm of heterophils and macrophages. Some alveolar parietal cells also exhibited positive signals (Fig. 6a). In contrast, caspase-3 positive signals from 1G1 group were restricted to cytoplasm of macrophages and heterophilic granulocytes, with no positive signals in alveolar parietal cells (Fig. 6b). No positive signals were observed in uninfected group (Fig. 6c). IOD (SUM) analysis showed that there was a significant difference between infected groups and uninfected group (Q vs uninfected: P = 0.0004; 1G1 vs uninfected: P = 0.0049; Fig. 6d), and a significant difference in expression level of caspase-3 between infected groups was also been noticed (1G1 vs Q: P = 0.0381; Fig. 6d). The results of western blot analysis were in congruence with the IOD analytic outcomes, showing that the highest level of caspase-3 (30 kDa) was expressed in Q group, which was significantly different from uninfected group and 1G1 group (Q vs uninfected: P = 0.0000; Q vs 1G1: P = 0.0000; Fig. 6f), while no statistical difference was found in 1G1 group compared with uninfected group. (P = 0.9681; Fig. 6f).

### Expression of TIMP1 and MMP9 in lung tissues of chickens after *P. multocida* infection

**Figure 7:**
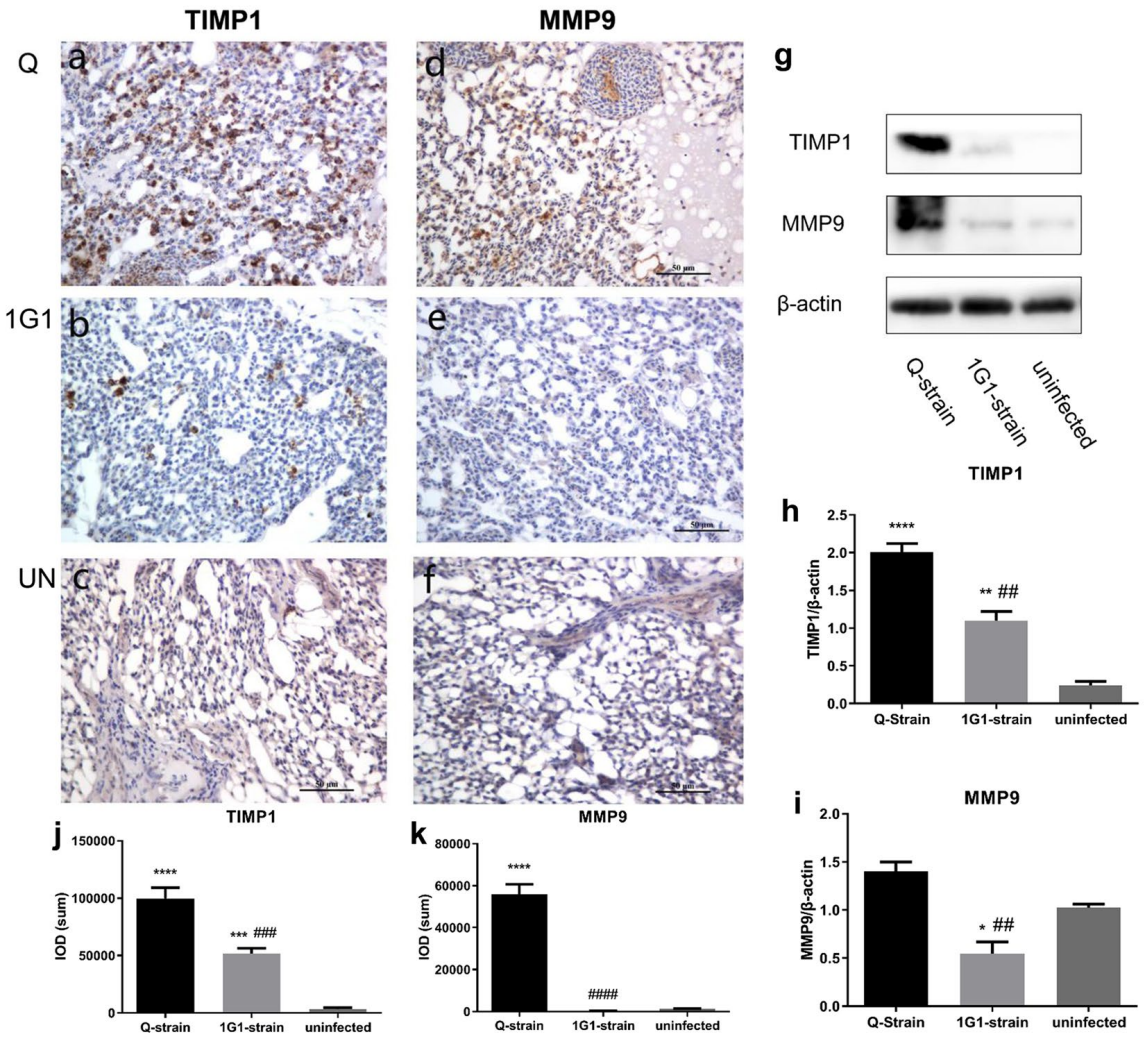
Expression of MMP9 and TIMP1 was significantly increased in infected chickens. (**a**–**f**) Immunohistochemical results of MMP9 and TIMP1. (**j**) and (**k**) Positive areas in the infected and uninfected groups were compared. (**g**) Representative western blot results of MMP9 and TIMP1. (**h**,**i**) A histogram of the expression of MMP9 and TIMP1 with β-actin as a control. UN, uninfected; Q, Q group; G, 1G1 group. (*P < 0.05; **P < 0.01; ***P < 0.001; ****P < 0.0001, compared with uninfected group; ^#^P < 0.05; ^##^P < 0.01; ^###^P < 0.001; ^####^P < 0.0001, compared with group G; n = 3 per group).

No TIMP1 expression was observed in uninfected lung tissues (Fig. 7c). However, TIMP1 was highly expressed in cytoplasm of heterophilic granulocytes and macrophages (Fig. 7a,b). The TIMP1-positive areas in 1G1 group were significantly different from Q group and uninfected group (1G1 vs uninfected: P = 0.0002; 1G1 vs Q: P = 0.0002; Fig. 7j), and the statistical difference between Q group and uninfected group was also significant (P = 0.0000; Fig. 7j). The western blot analytic results of TIMP1 (23 kDa) was consistent with the IOD (SUM) analysis, that is, the protein expression in Q group was significantly different from that in uninfected group and 1G1 group (Q vs uninfected: P = 0.0000; Q vs 1G1: P = 0.0017; Fig. 7h). The protein expression between 1G1 group and uninfected group was also significantly different (P = 0.0023; Fig. 7h). MMP9 was expressed in cytoplasm of alveolar parietal cells and smooth muscle cells in uninfected group (Fig. 7f). In contrast, low MMP9 expression was observed in lung tissues from 1G1 group (Fig. 7e), while Q group showed positive signals mainly in cytoplasm of heterophilic granulocytes and macrophages (Fig. 7d). The positive signal areas of Q group was significantly different from that of uninfected group and 1G1 group (Q vs uninfected: P = 0.0000; Q vs 1G1: P = 0.0000; Fig. 7k), but there was no significant difference between 1G1 group and uninfected group (P = 0.8912; Fig. 7k). The protein expression analysis of MMP9 (78 kDa) showed that the statistic result of Q group were significantly different from that of 1G1 group (P = 0.0015; Fig. 7i). However, there was no significant difference between Q group and uninfected group (P = 0.0614; Fig. 7i). The protein expression of 1G1 group was significantly different from uninfected group (P = 0.0252; Fig. 7i).

## Discussion

In the present study, we evaluated the histopathological changes in chickens infected with *P. multocida* and found that pathological changes in the infected group were mainly associated with inflammation. Unexpectedly, we did not observe significant necrotic foci in the lung tissue. However, pulmonary edema was very obvious. The occurrence of pulmonary edema indicated activation of the apoptotic FasL/Fas signaling pathway, activation of the renin-angiotensin signaling pathway, and activation of the NF-κB signaling pathway. Activation of these signaling pathways caused cell death and upregulates inflammatory factors that triggered inflammation^31,32^. Heterologous granulocytes were the main cells in infiltrating lung lesions, and the importance of heterophilic granulocytes in respiratory infections of *P. multocida* had also been confirmed. Heterologous granulocytes and other immune cells played a role in the development of infectious diseases in birds^33^. In this study, we found that the proteins studied were all expressed in heterophilic granulocytes. For example, RIPK was involved in LPS-induced proinflammatory responses, independent of MLKL, and was unaffected by it. In macrophages, the activity of RIPK1, RIPK3 and MLKL was reduced in the presence of caspase inhibitors following activation of Toll-like receptors (TLRs) 3 and 4^24^. In mice, LPS-induced RIPK1- and RIPK3-dependent inflammation did not require inhibition of caspase-8 activity^23^. Therefore, apoptosis might lead to RIPK1 and RIPK3-dependent inflammation. We found that macrophages expressed a large amount of these proteins, and the inflammatory process of chickens infected with *P. multocida* involved RIPK1 and RIPK3. In addition, analysis of caspase-3 localization and expression levels suggested that apoptosis might be the main pathway for avian cholera-induced lung injury. However, in terms of the results of MLKL, its expression was too low. These findings suggested that necrotic apoptosis might not be the main cause of lung damage in avian cholera. The expression of caspase-3 was the lowest in lung injury induced by 1G1 strain, suggesting that other forms of apoptosis were induced or independent of caspase-3 mediated cell death.

Apoptosis activated RIPK1 and RIPK3, further initiated the nuclear factor (NF)-κB signaling pathway and induced IL-6 secretion, thereby recruiting inflammatory cells and aggravating lungs via heterophilic granulocyte and macrophage infiltration and inflammation^11^. Due to cell death induced by *P. multocida*, the nuclear protein HMGB1 was transferred to the extracellular space, triggering an inflammatory response in the form of increased IL-6^34^. LPS from *P. multocida* stimulated activation of the nuclear factor kappa-light chain enhancer of the TLR4-mediated activated B cell (NF-κB) pathway, producing IL-6^35^.

When these proinflammatory cytokines were released and bound to receptors, the mitogen-activated protein kinase (MAPK) signaling pathway and NF-κB promoted the expression of cytokines and chemokines, thereby further activating immune responses and inflammation^36^. Inflammatory cells concentrated at the site of injury are stimulated by inflammatory factors and continued to release many proinflammatory factors, such as HMGB1 and IL-6, leading to cell death and potentiating inflammatory responses^37,38^. Thus, bacterial-induced cytokine secretion further mediated inflammatory responses in tissues, whereas bacteria associated with these cytokines or their products induced apoptosis of immunocompetent cells. Neutrophils could also increase the level of MMP9 released during early inflammation, resulting in enhanced inflammatory cell infiltration. TIMP1 would form a complex with MMP9 in a ratio of 1:1 to inhibit MMP9 activity^39^; however, the expression of TIMP1 in the experimental group was much higher than that of MMP9, probably due to its role in maintaining the microenvironment. Through the above studies, heterophilic granulocytes might play an important role in the infection against *P. multocida*.

In addition, the pathological changes caused by the two strains are also different. Considering that the 1G1 strain is a strain derived from avian cholera in a duck, its virulence may be impaired when it is transferred to a different host. According to previous studies in our laboratory, the 1G1 strain can induce typical symptoms of avian cholera in 20-day-old chicks, but the time points at which the 1G1 strain and the Q strain cause damage to the body are different. In chickens infected with the 1G1 strain, the expression levels of HMGB1 and MMP9 decreased, further indicating that the strain can inhibit inflammation, or that the 1G1 strain relies on a mechanism different from the Q strain to cause lung damage. Studies have shown that even in strains of the same serotype, the structure of LPS is very different, and the development of vaccines has had major difficulties^40^. Therefore, although the Q strain and the 1G1 strain are both serotype A, the virulence-determining LPS may still differ and induce different types of cell damage. Therefore, the pathogenic mechanism of the two strains needs further researches to confirm, especially the bacterial structure that determines the virulence of the two strains.

In summary, the proinflammatory response involving RIPK1 and RIPK3 suggests histopathological changes associated with heterophilic granulocytes that are associated with host response to *P. multocida* infection in the lung.

## Methods

### Experimental animals and ethics statement

Experimental animals was performed as described previously^41^. The rearing facilities and experiments in chickens were approved by the Research Ethics Committee at Huazhong Agricultural University (approval HZAUCH-2016-006). Chickens were housed indoors at a humidity of 50–60% and temperature of 25 °C, with free access to feed and drinking water. The feed was purchased from Wuhan Chia Tai Group (17.5 MJ/kg, 15% protein). All animal experiments were conducted in accordance with the recommendations of the Guide for the Care and Use of Laboratory Animals from the Ethical Committee for Animal Experiments at Huazhong Agricultural University, Wuhan, China. All efforts were made to minimize animal suffering and to reduce the number of animals used.

### Bacterial 16S rRNA and KMT1 genes amplification

Using an inoculating ring, the bacteria strains were applied to TSA medium and then placed in a 37 °C bacterial incubator for 24 h. Colony morphology was observed, and dominant colonies were carefully picked and then cultured on TSA medium at 37 °C for 18 h. A single bacterial colony was picked from the TSA plate and cultured in 5 ml TSB medium at 200 rpm and 37 °C for 12 h. After centrifugated at 12,000 rpm for 1 min, the supernatant fluid was discard. The preciptates were repeatedly washed with sterile 0.9% saline for 3 times to prepare bacteria samples. Bacterial DNA was purified by a EasyPure Genomic DNA Kit (TransGen Biotech, Beijing), and used as a PCR template. In order to amplify 16S rRNA and KMT1 genes, the two pairs of primers were used (Table 1)^42^. Other components of 25 uL PCR system includes 12 uL 2×EasyTaq^®^ PCR Super Mix (TransGen Biotech, Beijing), 1uL forward primer, 1uL reverse primer, 8 uL double-distilled water and 3 uL bacterial DNA template. Amplification was performed using an Applied Biosystems VeritiTM Thermal Cycler (Thermo Fischer Scientific, Waltham, MA, USA) with the following PCR conditions: initial denaturation at 94 °C for 3 min, 30 cycles of 94 °C for 30 s, 60 °C for 30 s, and 72 °C for 30 s, followed by a final extension at 72 °C for 10 min. Finally, all the PCR samples were analyzed by agarose gel electrophoresis (1% agarose, 120 V 15 min).

**Table 1:** Primer sequences.

Gene	Primer	Forward (5′-3′)	Reverse (5′-3′)	Accession no.	Length (bp)
16S rRNA	Pasteurella 16s	GAGTTTGATCMTGGCTCAG	CTAHAGGGTATCTAATCCT	NZ_CP008918	796
KMT1	KMT1	ATCCGCTATTTACCCAGTGG	GCTGTAAACGAACTCGCCAC	AF016259.1	460
hyaD-hyaC	cap-A	TGCCAAAATCGCAGTCAG	TTGCCATCATTGTCAGTG	AF067175	1044
bcbD	cap-B	CATTTATCCAAGCTCCACC	GCCCGAGAGTTTCAATCC	AF169324	760
dcbF	cap-D	TTACAAAAGAAAGACTAGGAGCCC	CATCTACCCACTCAACCATATCAG	AF302465	657
ecbJ	cap-E	TCCGCAGAAAATTATTGACTC	GCTTGCTGCTTGATTTTGTC	AF302466	511
fcbD	cap-f	AATCGGAGAACGCAGAAATCAG	TTCCGCCGTCAATTACTCTG	AF302467	851

### Baterial serotyping

Five specific primers were designed (Table 1) and multiplex serotyping PCR assay was used to identify the serotypes of *P. multocida*^43^. The 50 μL PCR system includes: 12 uL 2×EasyTaq^®^ PCR Super Mix (TransGen Biotech, Beijing), 1 uL for each primer, 2 μL baterial DNA templates, and 26 uL double-distilled water. Amplification conditions for PCR were as follows: initial denaturation at 95 °C for 5 min, 30 cycles of 94 °C for 30 s, 55 °C for 30 s, and 72 °C for 45 s, followed by a final extension at 72 °C for 10 min. PCR samples were analyzed using agarose gel electrophoresis (1% agarose, 120 V 15 min).

### *P. multocida* isolates and inoculum

Two strains of *P. multocida* were used: the standard strain Q (Chinese Veterinary Culture Collection Center CVCC44801) and the 1G1 strain. *P. multocida* 1G1 is a serogroup A strain isolated from the clinical disease in ducks and identified and preserved by Huazhong Agricultural University Pathology Laboratory. Cultures of *P. multocida* were grown in oscillation cultures in broth culture medium (Becton, Dickinson and Company, MD) at 37 °C. Samples for inoculation were diluted to a concentration of 10^4^ CFU/mL.

### Experimental design

The information for grouping and *P. multocida* infection in experimental animals was performed as described previously^41^.

### Histopathology and immunohistochemistry

Lung specimens were fixed in 10% neutral buffered formalin for 24 h, processed routinely, and embedded in paraffin wax. Tissue sections were stained with haematoxylin and eosin (HE) and subjected to immunohistochemistry (IHC).

For IHC, sections were mounted on polylysine adhesive slides (ZSGB-BIO, Co., Ltd., Beijing, China). Slides were heated to 60 °C, deparaffinized and rehydrated by passage through xylene and graded concentrations of ethanol. After the slides were washed in distilled water, they were heated for antigen retrieval and treated with endogenous peroxidase blocking solution (ZSGB-BIO). The sections were then incubated with rabbit anti-human caspase-3 antibodies (CSB-MA080226; CusAb, Wuhan, China), rabbit anti-RIPK1 polyclonal antibodies (A7414; Abclonal, Wuhan, China), rabbit anti-RIPK3 polyclonal antibodies (A5431; Abclonal), anti-MLKL polyclonal antibodies (CSB-PA003250; CusAb), rabbit anti-MMP9 polyclonal antibodies (A0289; Abclonal), rabbit anti metalloproteinase inhibitor 1 polyclonal antibodies (CSB-PA024013YA01HU; CusAb), rabbit anti HMGB1 polyclonal antibodies (CSB-PA01604A0Rb; CusAb), or rabbit anti IL-6 polyclonal antibodies (CSB-MA067571A0m; CusAb) diluted 1:200 in 5% bovine serum albumin (BSA). We also used mouse anti-*P. multocida* antibodies (a gift from Professor Wu Bin, National Key Microbiology Laboratory, Huazhong Agricultural University). The bacterium for antibody preparation was a strain of *P. multocida* HB03 isolated from a pig that was also type A and diluted at 1:400 in 5% BSA. The sections were incubated with phosphate-buffered saline (PBS) in a refrigerator at 4 °C overnight. The sections were incubated with enhancer (ZSGB-BIO) for 20 min at 25 °C. Polymerized horseradish peroxidase (HRP)-conjugated goat anti-rabbit/mice immunoglobulin (Ig; ZSGB-BIO) was applied to the sections for 30 min, followed by two washes in PBS. The sections were then placed in DAB for approximately 5 min. After washing with distilled water, haematoxylin (ZSGB-BIO) staining was performed for 10 s and sections were washed with hydrochloric acid and tap water. After deparaffinization and rehydration, the sections were mounted under glycerol-gelatine. Negative controls for IHC were produced by substituting the primary antibody with BSA. The sections were evaluated by light microscopy (Nikon, Japan). We set the amount of the target protein based on the density of the dye color and the size of the distribution area. The total amount of target proteins is proportional to its stained distribution areas, because the quantitative relationship between the color depth of the stain and the target protein amount is in accordance with Lambert-Beer’s law and is a logarithmic relationship.

After the immunohistochemical staining was completed, we randomly selected three slices from each group and five different fields of view per slice, at 400×. The intensity of the positive signal after staining was quantitatively analyzed using Image-Pro Plus analysis system (Media Cybernetics, Inc., San Diego, CA). The specific method is:

First, the optical density value is corrected. The white color with a large gray value is defined as the optical density value equal to 0, and the optical density value of the pure black dot is set to 3.0. The conversion relationship between the optical density value and the gray value is in accordance with Lambert-Beer law, so they are logarithmic relations.

After that, we selected specific objects to be tested. The yellow areas in the immunohistochemical slices in our study were positive staining areas. Use the segmentation tool in IPP to automatically select the yellow areas in photos.

Then, the IOD (integrated optical density) of all selected objects are accumulated to obtain an accumulated value (IOD SUM). Each photo gets a measurement.The measurements of the same slices were averaged, and then a total of three slices per group were used to calculate the mean and standard deviation, and one-way ANOVA was used to analyze the significant differences between the groups.

As for taking photos of these samples, we adjusted the microscope light to about 9 V and the camera exposure time, and finally made the background appear white. The gray value reached 230 or more. Use the manual correction white balance function to correct the background color to pure white. All photos were taken at once using the same microscope operating conditions and camera operating conditions (exposure and white balance settings). Save the photo as TIFF format.

### Protein extraction and western blotting

Protein extraction and western blotting were performed as described previously^41^. Briefly, we applied the methods reported previously to detect expression of the following proteins in the lung: RIPK1, RIPK3, IL-6, HMGB1, caspase-3, TIMP1 and MMP9. The specific information on materials we employ in this study is the same as previously reported.

### Statistical analysis

Statistical analysis was performed as described previously^41^.

## Supplementary information

Supplementary information.

## Data Availability

All data generated or analysed during this study are included in this published article and its Supplementary Information file.
