# Supplementary material for: Receptor-interacting serine/threonine kinase 1- and 3-dependent inflammation induced in lungs of chicken infected with Pasteurella multocida
Source: Sci Rep. 2020 Apr 14;10:6340. doi: 10.1038/s41598-020-62042-7 (PMC7156477; doi:10.1038/s41598-020-62042-7)
Supplement: Supplementary file 1 — Supplementary information. [file 41598_2020_62042_MOESM1_ESM.pdf]

Receptor-interacting serine/threonine kinase 1- and 3-dependent inflammation induced in lungs of chicken infecteded by Pasteurella multocida

Weitian Li•, Qiyu Tang•, Na Dai, Weikuan Feng, Changqing Xie,Guofu Cheng，Xiaoli Liu， Wanpo Zhang，Xueying Hu，Changqin Gu*

1 Department of Basic Veterinary Medicine, College of Veterinary Medicine, Huazhong Agricultural University, Wuhan, Hubei 430070, China

●: These authors contributed equally to this work.

*Corresponding author: Department of Basic Veterinary Medicine, College of Veterinary Medicine, Huazhong Agricultural University, No. 1 Shizishan Street, Hongshan District, Wuhan 430070, P.R. China. Tel.: +86-155-2783-6768; E-mail: guchangqin@mail.hzau.edu.cn

Supplementary information:

We added the full-length gels and blots and multiple exposures in this supplementary information.

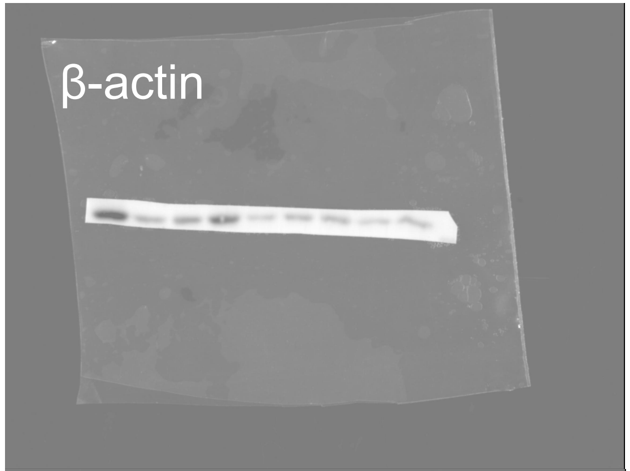

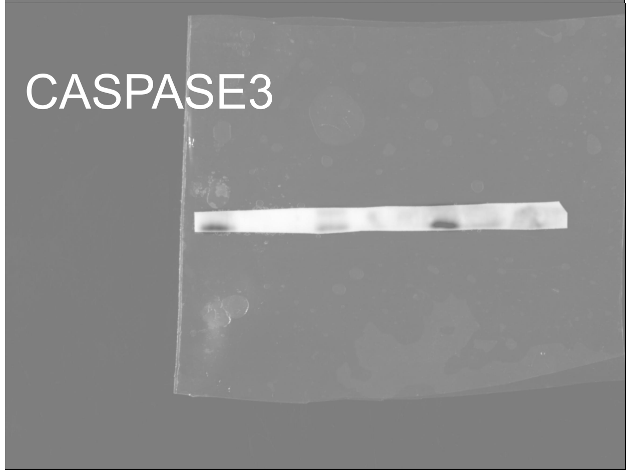

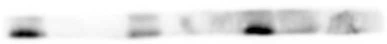

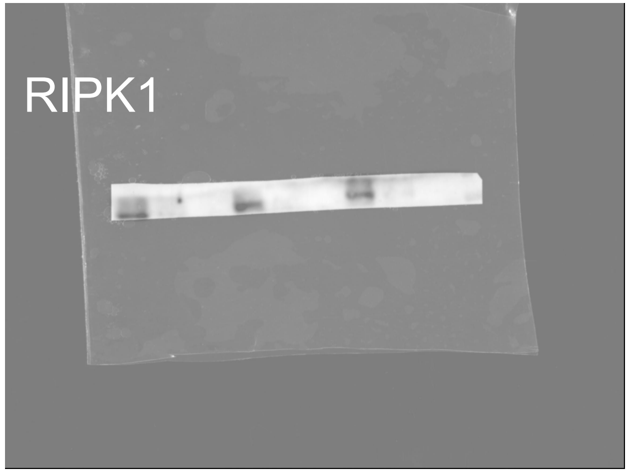

2017-02-25

β-actin

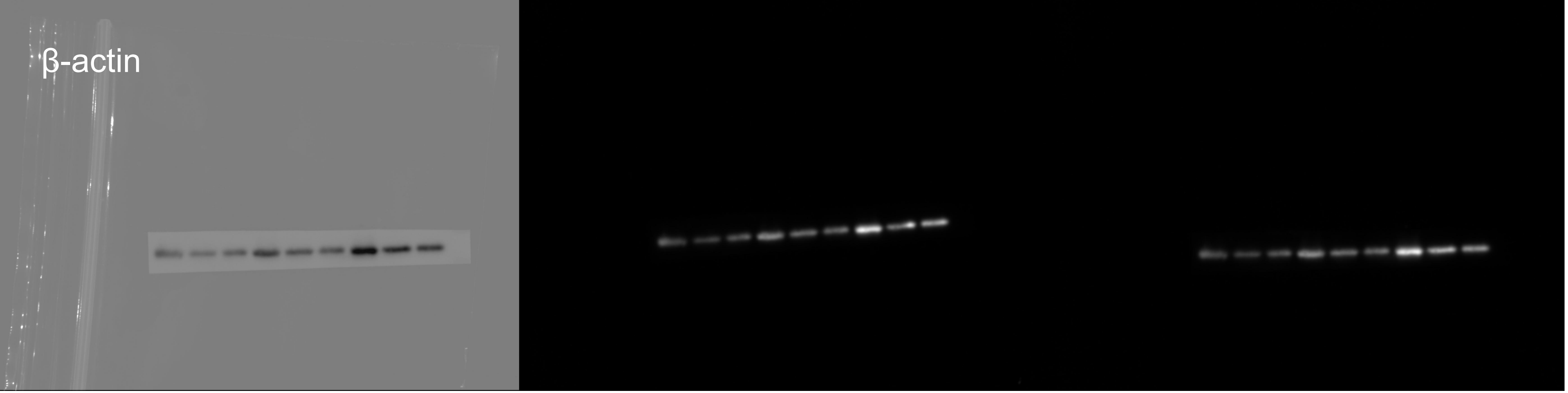

TIMP1

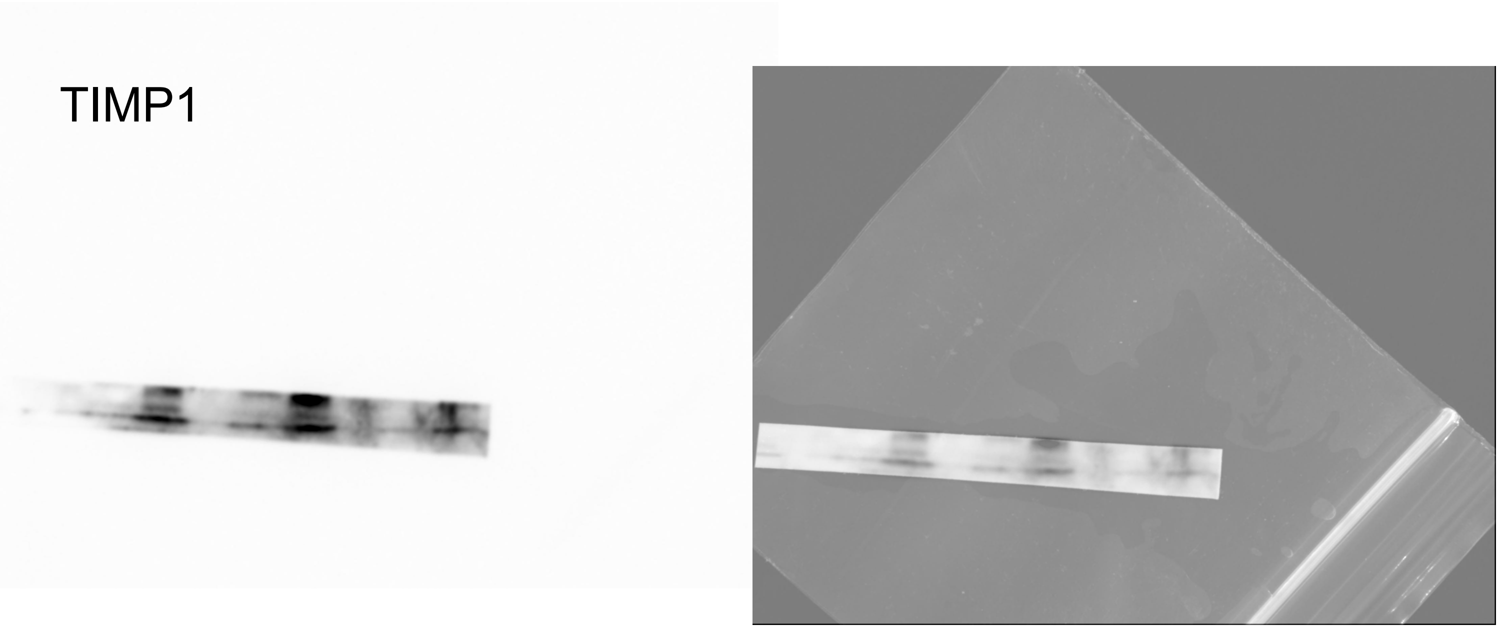

2017-02-27

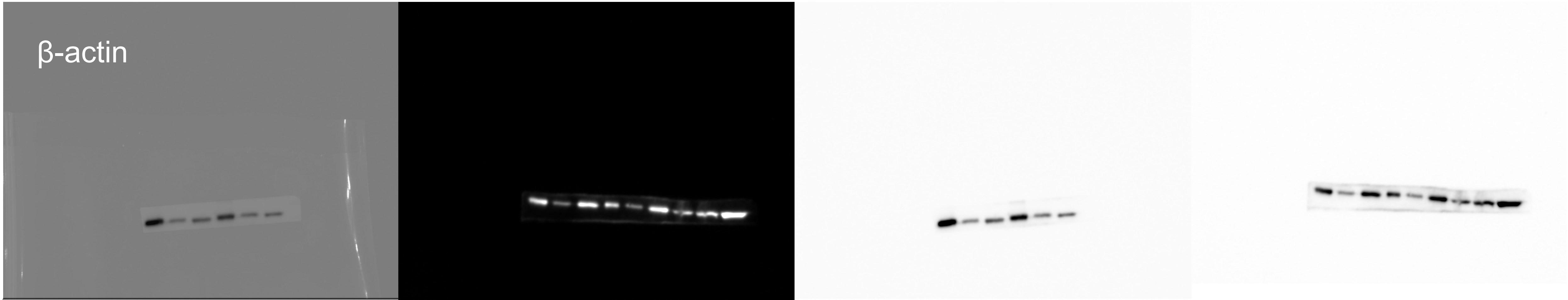

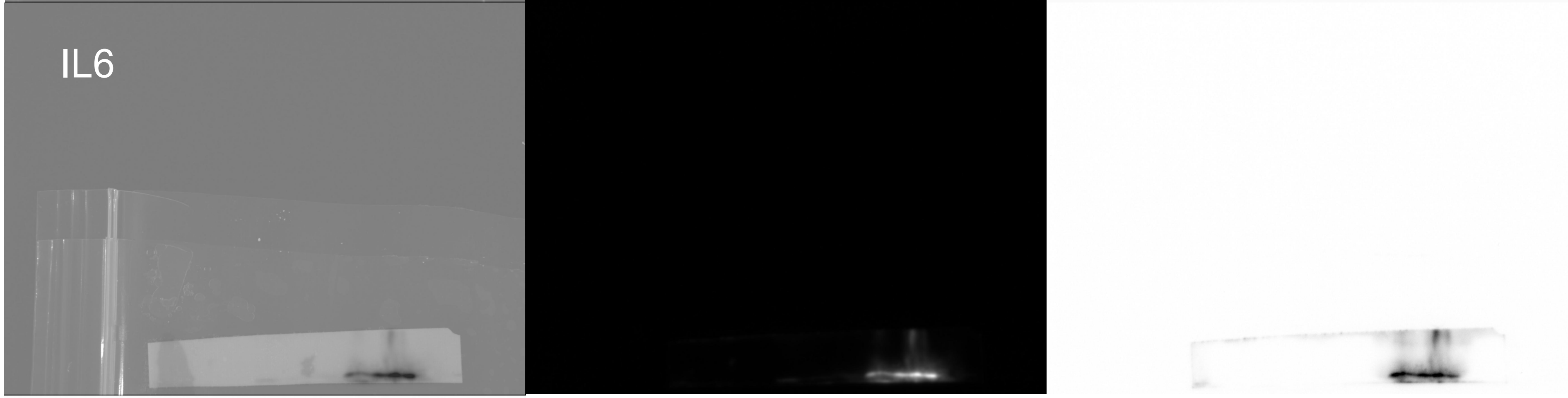

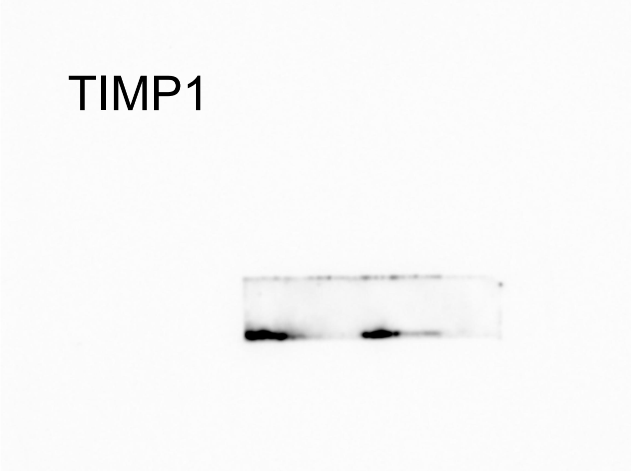

2017-03-03

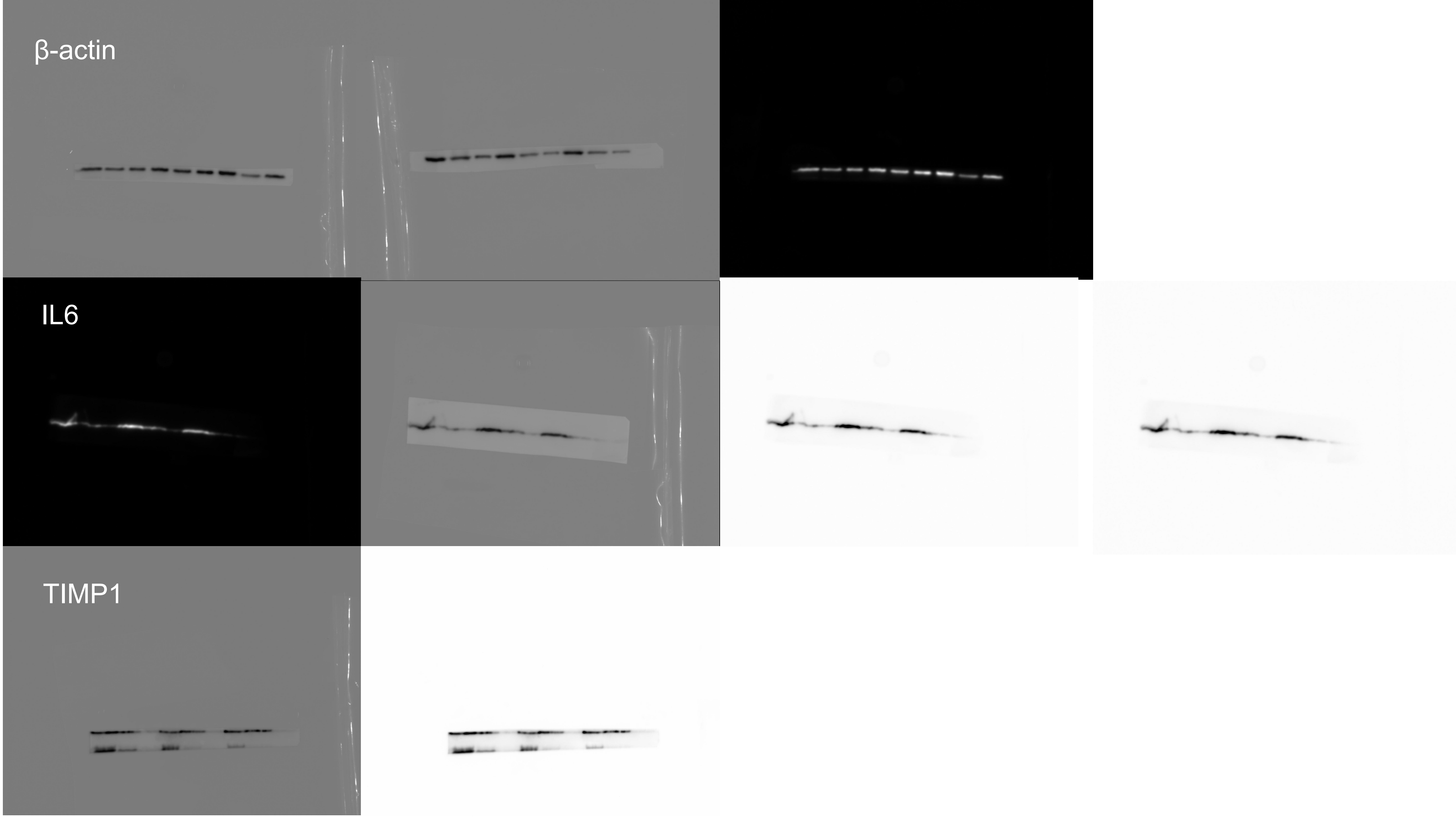
β-actin
IL6
TIMP1

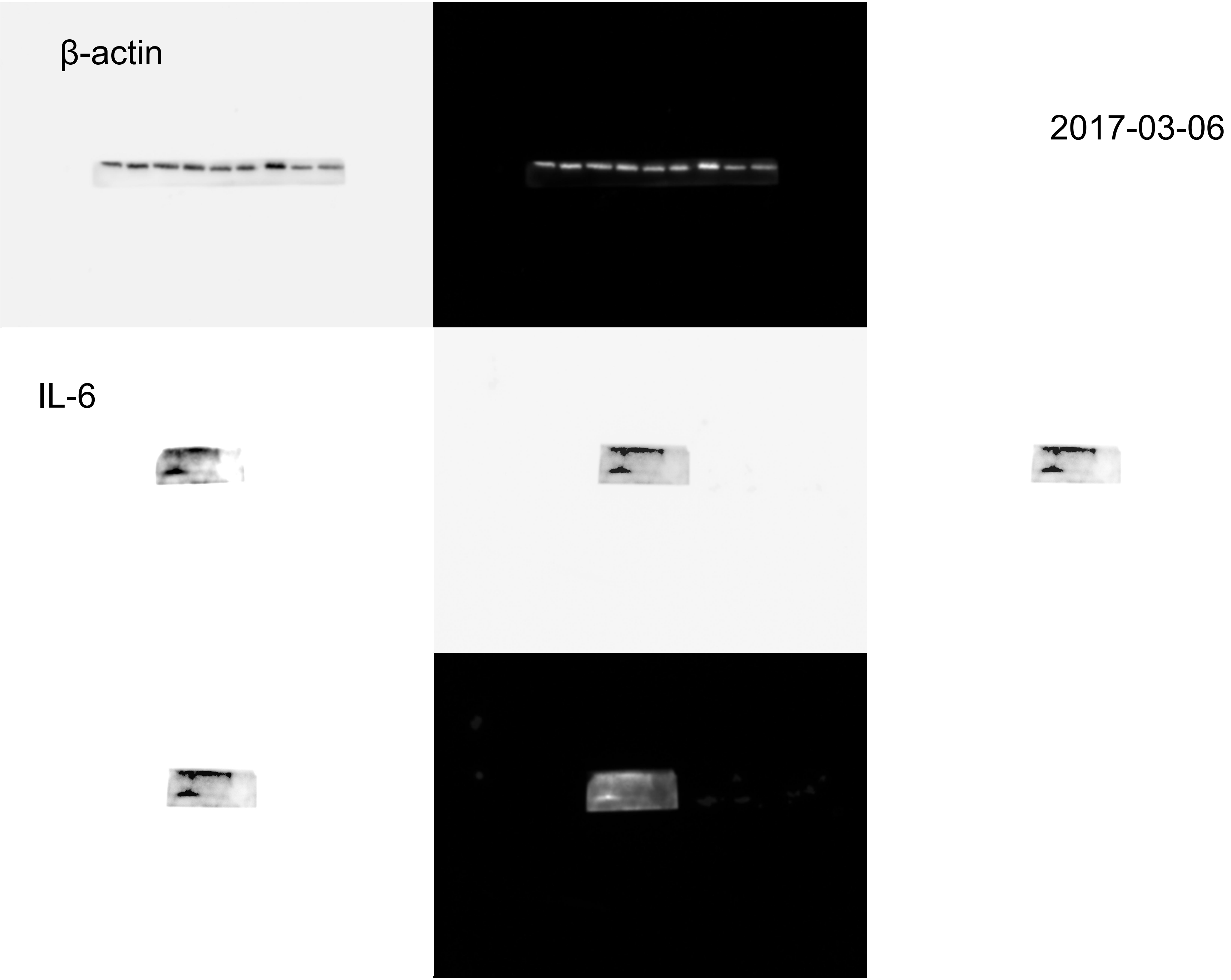
β-actin
2017-03-06
IL-6

2017-03-06

MMP9

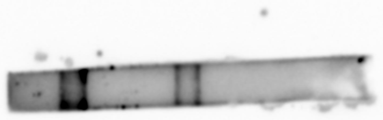

RIPK3

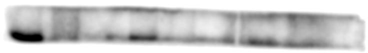

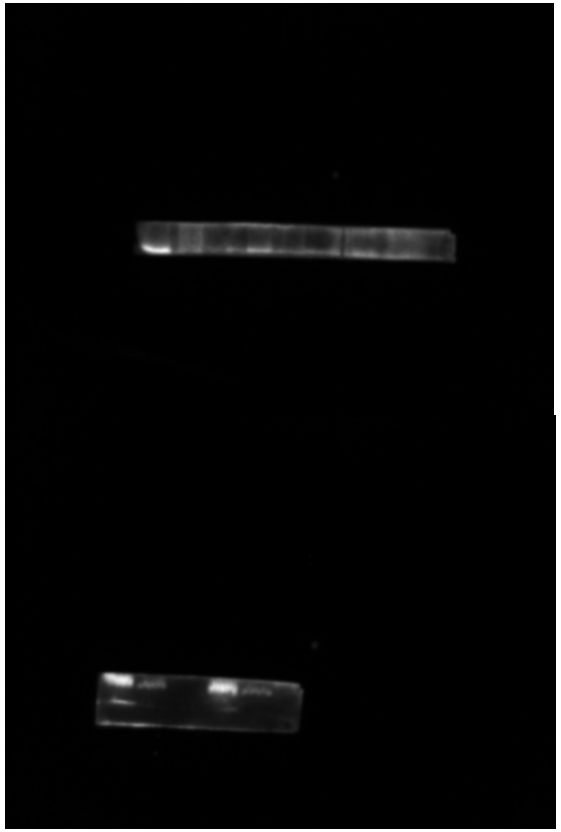

TIMP1

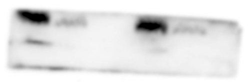

β-actin

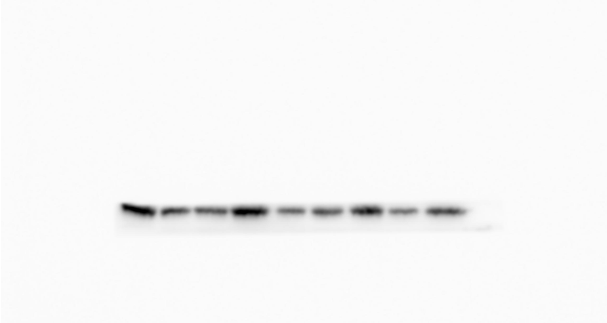

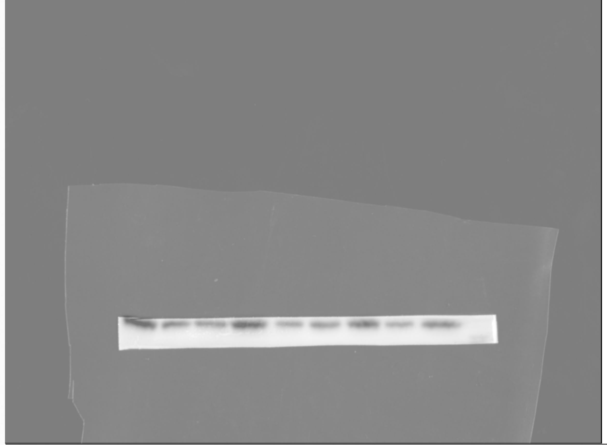

2017-03-10

MMP9

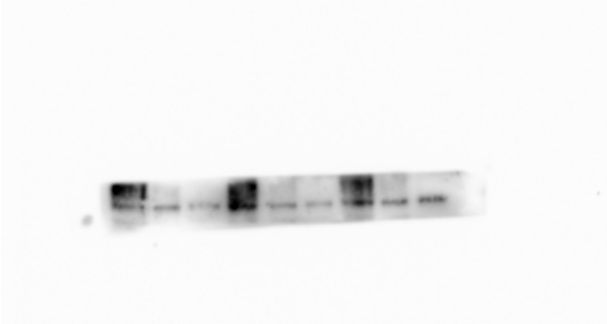

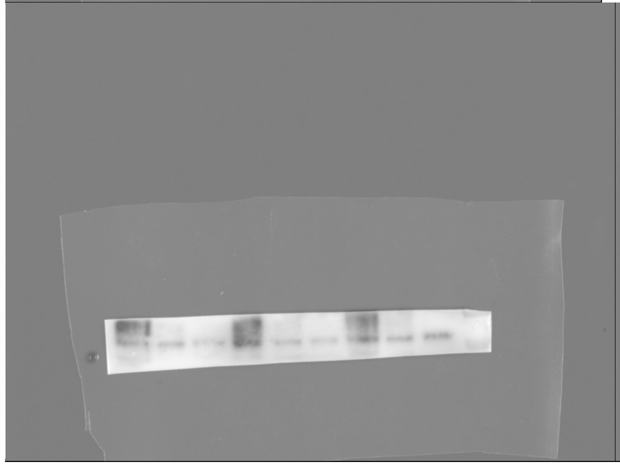

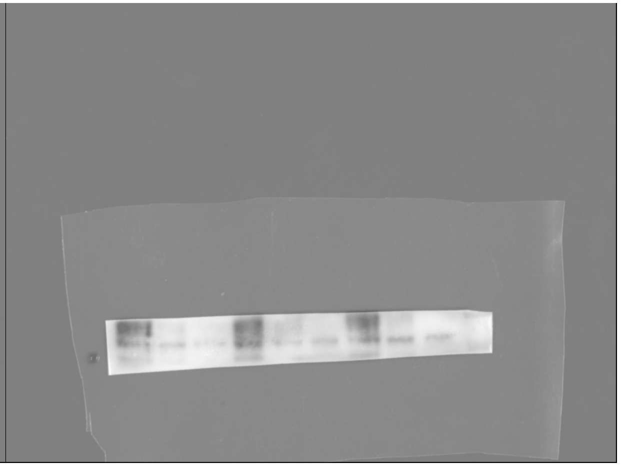

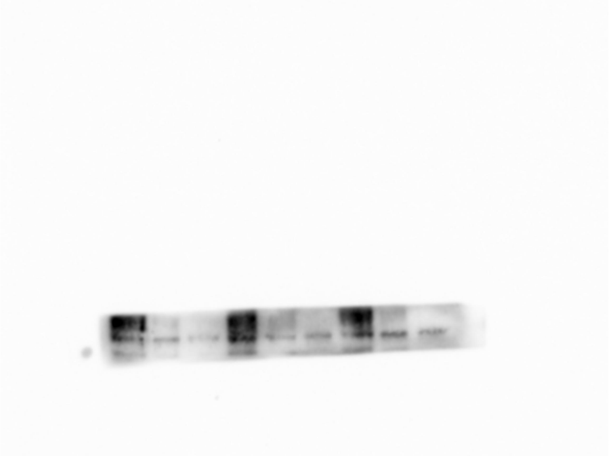

RIPK3

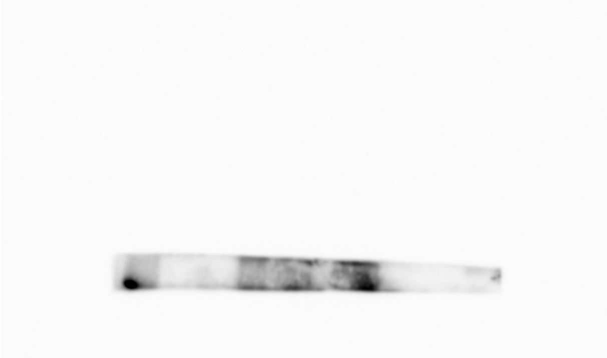

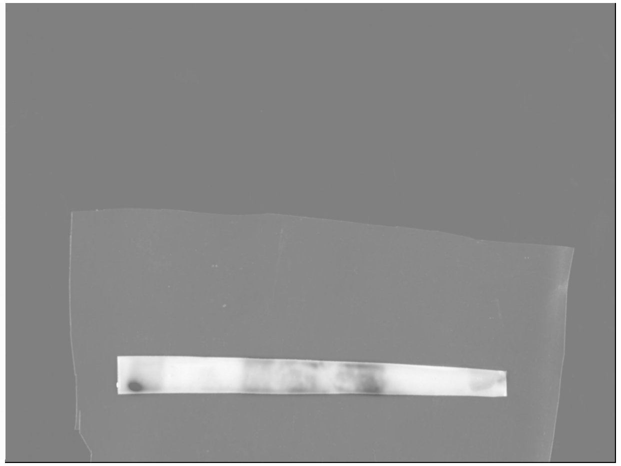

β-actin

2017-03-11

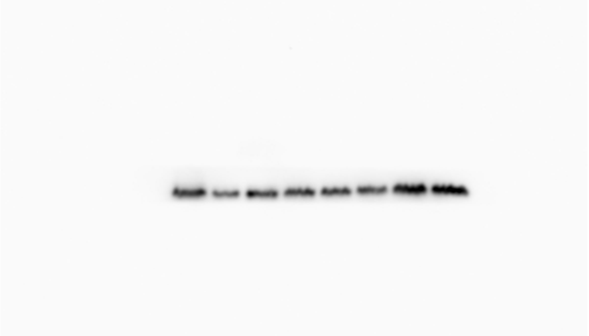

RIPK1

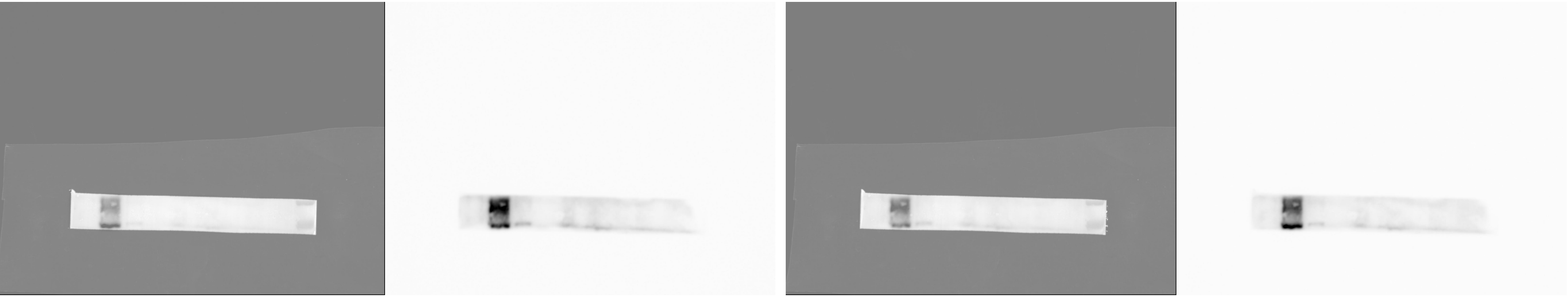

β-actin

2017-03-16

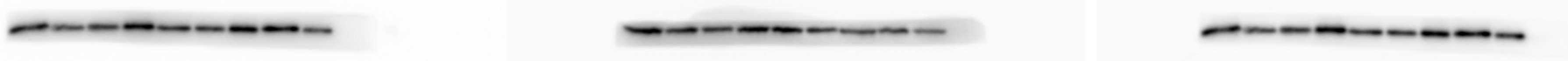

HMGB1

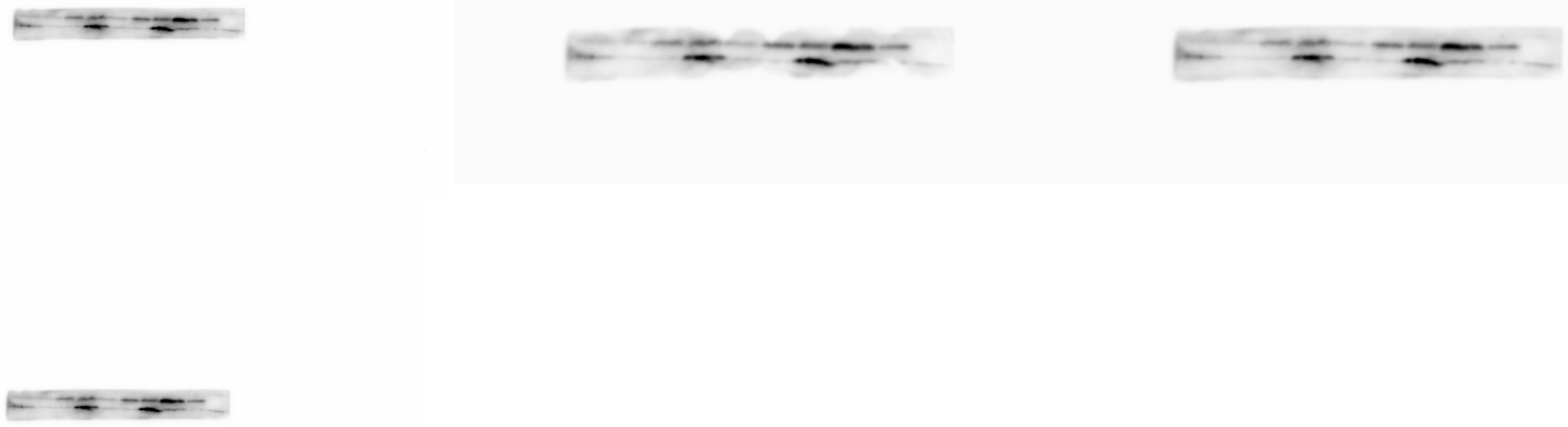

β-actin

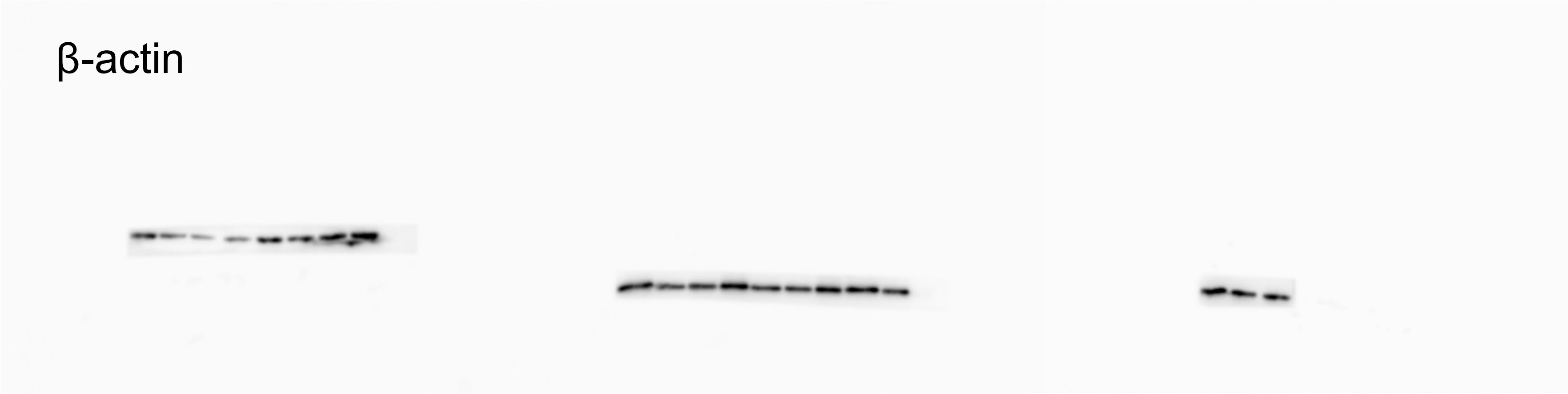

2017-03-16

MMP9

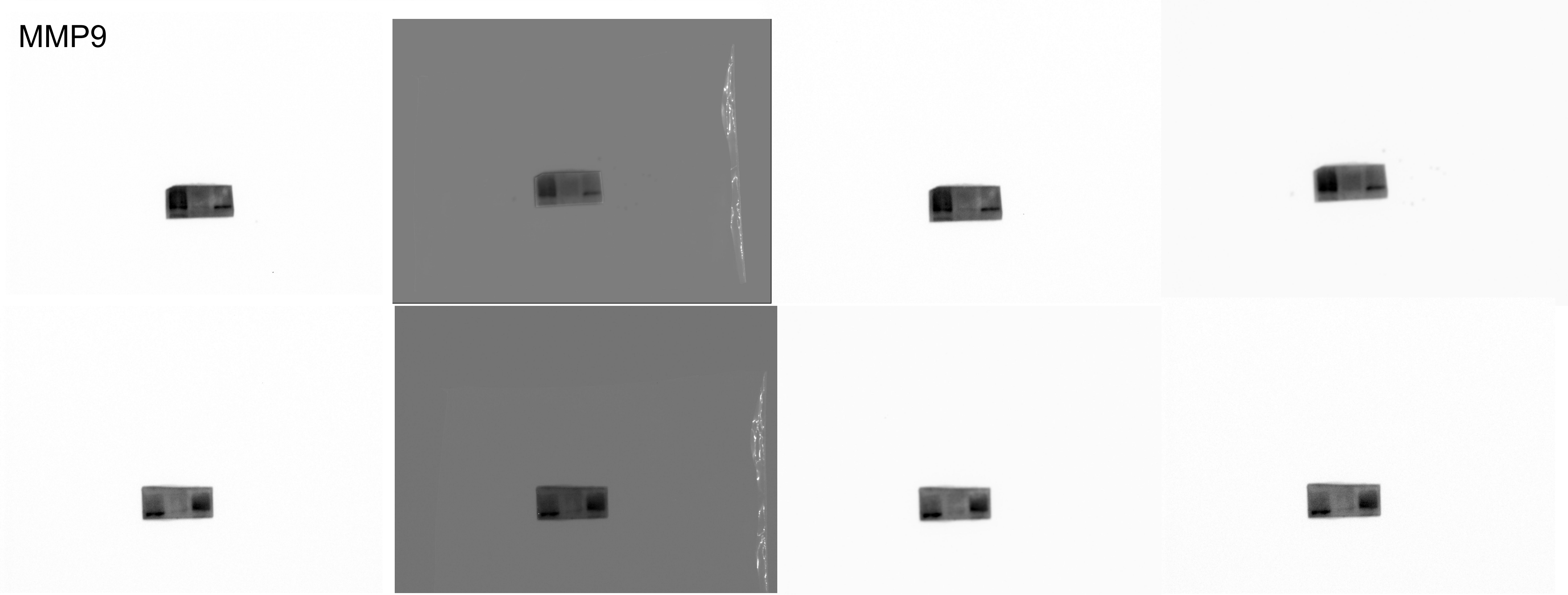

β-actin

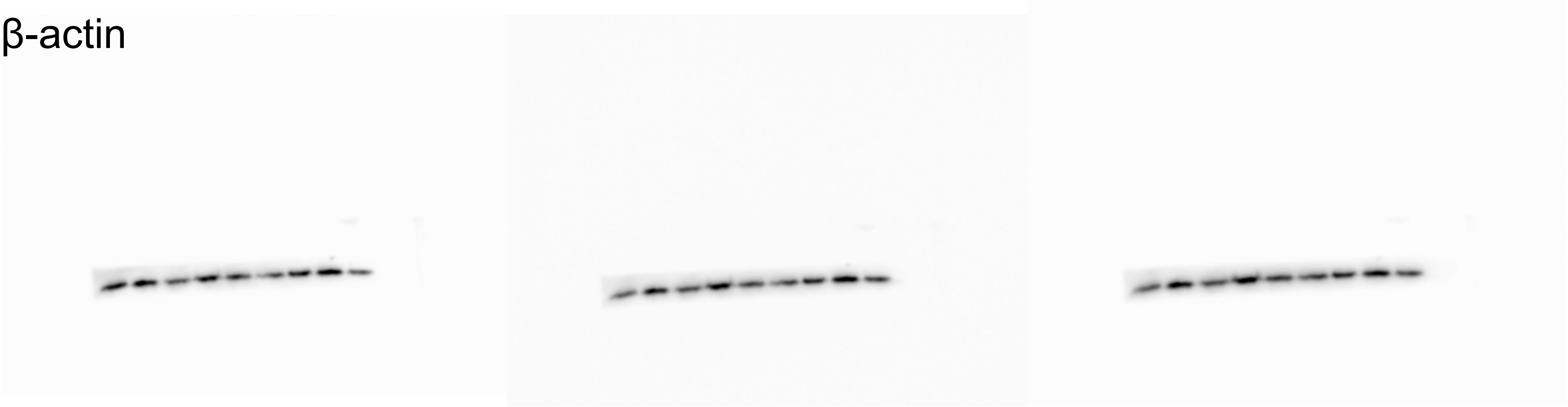

HMGB1

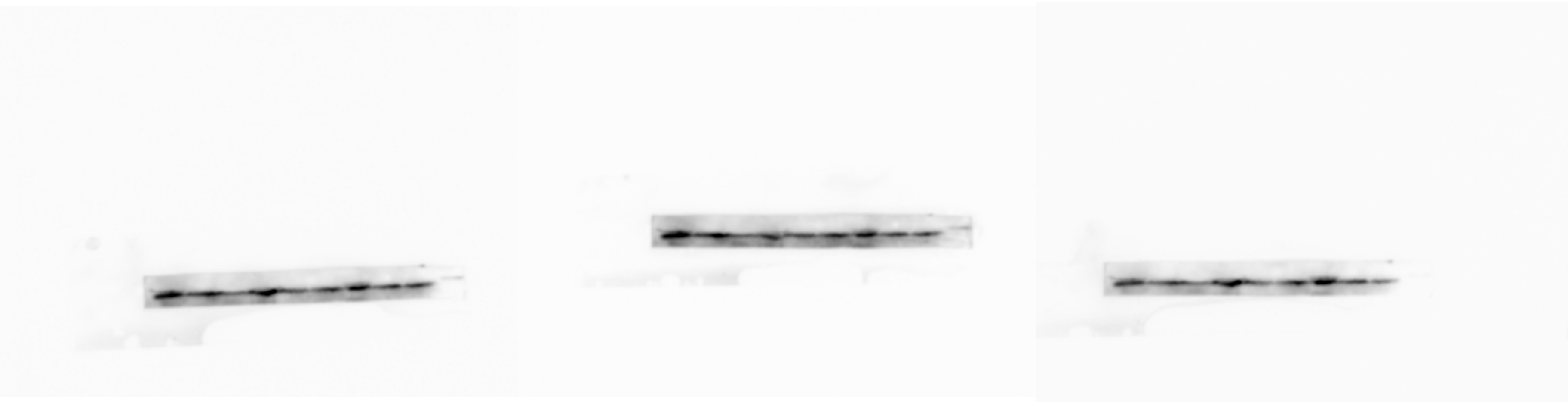

2017-03-19
